# Pyrrolizidine Alkaloids in the Food Chain: Is Horizontal Transfer of Natural Products of Relevance?

**DOI:** 10.3390/foods10081827

**Published:** 2021-08-07

**Authors:** Mohammad Said Chmit, Gerd Horn, Arne Dübecke, Till Beuerle

**Affiliations:** 1 Institute of Pharmaceutical Biology, Technical University of Braunschweig, Mendelssohnstr. 1, 38106 Braunschweig, Germany; s.chmit@tu-bs.de; 2 Exsemine GmbH, Am Wehr 4, 06198 Salzatal, Germany; g.horn@exsemine.de; 3 Quality Services International GmbH, Flughafendamm 9a, 28199 Bremen, Germany; arne.duebecke@tentamus.com

**Keywords:** biodegradation, pyrrolizidine alkaloid (PA), contamination, food chain, compost, plant waste, *Lappula squarrosa*, *Senecio jacobaea*, HPLC-ESI-MS/MS, horizontal alkaloid transfer

## Abstract

Recent studies have raised the question whether there is a potential threat by a horizontal transfer of toxic plant constituents such as pyrrolizidine alkaloids (PAs) between donor-PA-plants and acceptor non-PA-plants. This topic raised concerns about food and feed safety in the recent years. The purpose of the study described here was to investigate and evaluate horizontal transfer of PAs between donor and acceptor-plants by conducting a series of field trials using the PA-plant *Lappula squarrosa* as model and realistic agricultural conditions. Additionally, the effect of PA-plant residues recycling in the form of composts or press-cakes were investigated. The PA-transfer and the PA-content of soil, plants, and plant waste products was determined in form of a single sum parameter method using high-performance liquid chromatography mass spectroscopy (HPLC-ESI-MS/MS). PA-transfer from PA-donor to acceptor-plants was frequently observed at low rates during the vegetative growing phase especially in cases of close spatial proximity. However, at the time of harvest no PAs were detected in the relevant field products (grains). For all investigated agricultural scenarios, horizontal transfer of PAs is of no concern with regard to food or feed safety.

## 1. Introduction

**Figure 1:**
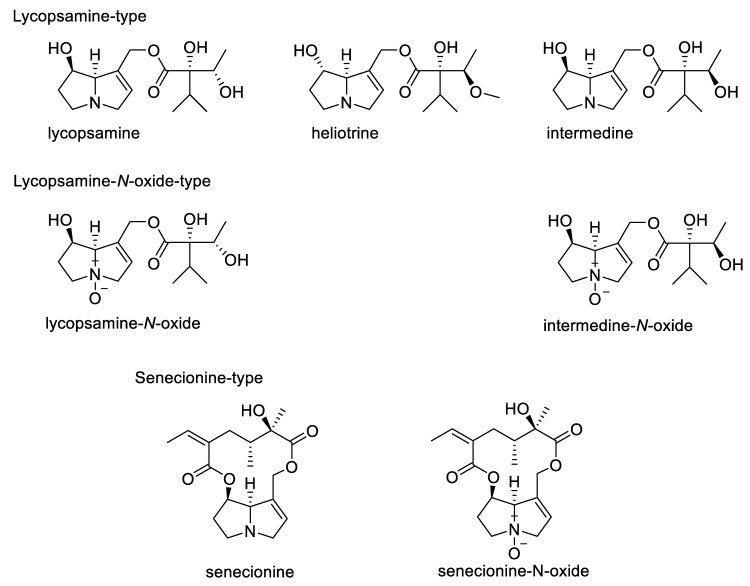
Exemplary chemical structures and features of some pyrrolizidine alkaloids (PAs).

To date, many cases of human and livestock poisonings are recorded in literature as a result of food, phytomedicines, or feed contamination with secondary metabolites of toxic plants such as pyrrolizidine alkaloids (PAs), cardiac glycosides or other toxic compounds [1,2]. In the case of PAs, these toxins are introduced mainly by accidental co-harvesting of poisonous PA plants together with the crop of interest [3]. Pyrrolizidine alkaloids (PAs) are a class of phytotoxins occurring in an estimated 3% of the flowering plants worldwide [4,5,6]. Presently, more than 660 individual PAs and PA-*N*-oxides (PANOs) have been structurally characterized [4], and three plant families (Asteraceae, Boraginaceae, and Fabaceae) are by far the most important sources of these toxins [4,6]. In the case of the Asteraceae, PAs occur mainly in the tribes Senecioneae and Eupatorieae. Within the Fabaceae, the genus Crotalaria is well known for PAs, while PAs are common in many genera of the Boraginaceae family [4]. Most derived PAs from plants can be assigned to one of four main structural classes and in particular, 1,2-unsaturated ester-PAs (see Figure 1), have been associated with hepatotoxicity and carcinogenicity [7,8].

The increasing awareness of PA-toxicity and the elucidation of the corresponding mode of action of PA-toxicity over the past few years, has triggered studies on various routes on how these toxic plant metabolites may enter and contaminate food and feed chains, although (with only a few exceptions) PA-plants are not used for crop production [9,10,11].

So far, accidental or unwanted co-harvesting has been identified as the main source of PA-contamination potentially harmful to humans or livestock as a result of mixing leaves of plants that contain PAs with leaves of medicinal herbs or teas [12] or of mixing the seeds of PA-plants with wheat and barley crops [13]. Commercial fraud and mixing medicinal herbs with other plants to meet price, volume, or other market demands, might be another source for this contamination [2]. In addition, flowers, with their contents of nectar and pollen, are by now a well-known cause of food contamination with PAs [14]. Many studies have been conducted on beehive products (honey, royal jelly, pollen food supplements) and have demonstrated that these products could naturally be contaminated by PAs [14]. When tracing the sources of contamination, it was found that nectar of PA-producing plants was the main source of contamination of these beehive products which could additionally increase by the PA-load of pollen from those flowers [14,15]. Furthermore, animal products can be also contaminated with PAs [12]. As stated, eggs, milk, cheese, and at low levels, meat, were shown to contain PAs if the feed of animals contained PA-plants in the first place [10]. 

Recently, a new path via horizontal PA-transfer of PA-plants or decaying PA-plant material to neighboring non-PA-plants was proposed and discussed as an additional source of PA-contamination of non-PA-crops [16]. Similar observations are known for xenobiotica such as insecticides, polycyclic aromatic hydrocarbons (PAH), pentafluorophenol or pharmaceutical products/metabolites, which are transferred directly from contaminated soil to plants via the roots [17,18,19]. However, such transfers are less known and so far, less studied for natural compounds and plant-to-plant transfer. Since chronic intake of low amounts of PAs may be potentially hazardous to humans and livestock [20], the contribution of each possible route of PA-entry (e.g., honey, tea, spices, etc.) should be carefully investigated to evaluate the importance of its contribution to the overall risk of PAs in the food chain. So far, horizontal PA-transfer was demonstrated under laboratory or laboratory-like conditions only, demonstrating the possibility of a transfer of PAs from mulched PA-plants to non-PA-plants as well as, e.g., from *Senecio jacobaea* to various herbs such as mentha or chamomilla [21], or between living plants in close spatial proximity, e.g., from *S. jacobaea* to various herbs like parsley or melissa [22]. In addition, a recent published study reported the transfer from strongly PA-plant infested fallow land (*Chromolaena odorata*) after slash-and-burn and slash-and-mulch practice to the subsequently cultivated maize plants [23]. These studies have in common that the transfer is usually observed from a PA-weed to crops under a more or less extreme PA-plant excess (potted plant experiments or PA-plant invasions). These conditions are usually not found in Western agricultural economies; hence these findings hardly mirror the potential impact to evaluate a realistic risk of horizontal PA-transfer in agricultural practice. So far, the ratio of non-PA-plant to neighboring PA-plants or decaying PA-plant material was not reflecting a realistic agricultural practice, where PA-plants/PA-biomass (here: weeds) should be by far outnumbered by the cultivated non-PA-crop plants. In addition, climate, soil, and seasons should have a major impact on the transfer rate (e.g., in the majority of cases the crop is harvested before PA-plants start to accumulate or decay). Furthermore, while in previous model experiments higher PA-levels were detected in roots and leaves/stems, in many realistic scenarios, seeds would be the final crop/product, but so far only very little data is available here [23]. In order to gain better insight into how these above-mentioned second-degree contamination routes impact the PA-contamination of non-PA-crops, we decided to conduct field experiments for several seasons to investigate the scenario of horizontal PA-transfer under realistic, actual agricultural and climate conditions.

## 2. Materials and Methods

### 2.1. Field Experiments

Field trials were carried out in accordance with the general guidelines of field trials methodology [24] in the time period between 2017 to 2020, in the surroundings of Halle/Saale located in the rain shadow of the Harz Mountains range (“Mitteldeutsches Trockengebiet”, Central German Dry Area). This consists of a dry but fertile landscape in the center of Germany close to Halle/Saale (Saxony-Anhalt, Germany). The altitude there is approximately 100 m above sea level. The mean for the long-term annual precipitation in this area is below 500 L/m^2^, and the mean annual temperature is 9.0 °C [25]. 

However, comparing the vegetation periods between 2018 and 2020, 2018 was considerably drier (<400 L/m^2^), whereas other locations showed even lower measurements (<300 L/m^2^). All three periods were significantly warmer following the general trend of the climate changes that took place there (annual mean values of up to >11 °C during the trial periods).

**Figure 2:**
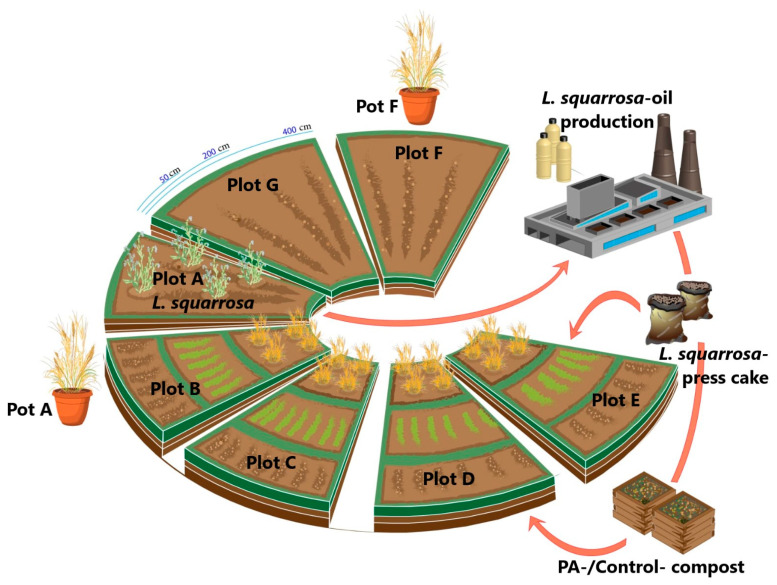
Experimental design to investigate possible horizontal PA-transfer in the context of PA-plant (*L. squarrosa*) cultivation.

All soils of the experiments had a medium to high credit rating (diluvial and loess sites, soil value indices, “Ackerzahl” [26]) of 54–98 and had a medium to elevated level of macro and micronutrient supply. All field experiments and the control plots were managed ecologically during these experiments and were in line with good agricultural practice methodology, while weed control was carried out exclusively by mechanical means. For the cultivation of *L. squarrosa* only the variety “Laira” was used. This is a biennial genotype, which is cultivated during autumn sowing of the previous year. The crops grown after *L. squarrosa* cultivations were chosen based on their importance for a rational crop rotation scheme for this region and for organic farming, constituting the idea that different possible plant families should be represented in these experiments. Regionally common varieties were used for these subsequent cultivations. Several scenarios of field experiments were conducted and the details are described below. In addition, Figure 2 in Results and Discussion illustrates and summarizes the chronology and rotation of field and crops.

### 2.2. Soil Improvement Measures

#### 2.2.1. Composts

Composts were prepared in 2018 and 2019 (July through September) to yield four different model composts which were used to test the impact of PA-plant compost on PA-transfer to subsequent crops: (a) control-compost contained only compost stock; (b) Senecio-compost contained compost stock of which 32% (*w*/*w*) of fresh cut *S. jacobaea* material were included; (c) Lappula-compost contained compost stock of which 14% (*w*/*w*) *L. squarrosa* press-cake powder was included; (d) Senecio/Lappula-compost contained both 15% (*w*/*w*) fresh cut *S. jacobaea* and 9% (*w*/*w*) *L. squarrosa* press-cake powder in the composite. 

Details on the compost making, PA-degradation and final PA-levels of the composts are given in Chmit et al. [27]. These four composts were analyzed for value-determining soil nutrients [27].

#### 2.2.2. *L. squarrosa* Press-Cake

Dried *L. squarrosa* press-cake (residues of the pressed *L. squarrosa* seeds for oil production; harvest 2017) was used in homogenous milled form (3 mm) provided by Exsemine GmbH (Salzatal, Germany). Samples were taken to analyze the PA-content of the press-cake before it was used to set up the composts (2018 and 2019) and before the material was added directly to the soil for field plot preparation in 2019 and 2020.

### 2.3. PA-Transfer via PA-Plant Compost/Press-Cake Using Experimental Field Plots 

Compost/press-cake experiments were performed in 2018/2019 in Etzdorf (Saxony-Anhalt, Germany) on a single field plot with a high-quality a high-quality loess-loam No.1 soil (“chernozem”, soil value index 98). The area was divided in individual 1.7 m × 1.7 m plots with a 2 m distance to each other in all directions. Six different cultivation experiments were investigated: control-compost, Senecio-compost, Lappula-compost, Senecio/Lappula-compost, direct *L. squarrosa* press-cake, and mineral fertilizer as a control plot. Two crops were used as potential acceptor-plants: winter wheat (*Triticum aestivum*), variety “Wiwa” and summer barley (*Hordeum vulgare*), variety “Avalon”. Four replicates of each treatment were conducted, resulting in 48 individual plots that were managed according to good agricultural practice (Figure 4). The corresponding amounts of compost/press-cakes were added to each plot to meet the nutrient requirements of the crop to be grown, based on total nitrogen content (*T. aestivum* 100 kg/ha and *H. vulgare* 50 kg/ha). A table showing the nutritional content and the added amount per plot is given in the Supplementary Materials file (Table S1).

The corresponding amounts of 2018 composts/press-cake quantities were added and worked in, on 18 October 2018 for both crops. This was done manually to avoid cross contamination between different plots. Winter wheat was sown on 19 October 2018 and spring barley in 4 March 2019, using a row distance of 25 cm. The mineral fertilization of the control plot was carried out for winter wheat on 26 April 2019, and for spring barley 23 May 2019 (at the two-node stage). 

A second, very similar experiment was conducted in 2019/2020, in the area of Zappendorf (Saxony-Anhalt, Germany) near a high-quality, loess-loam No. 2 site (soil value index 85). The analog experiment had the same variants. However, due to technical/agricultural reasons, instead of 4 replicate plots for each treatment larger individual plots were used (3 m × 5 m) to allow similar numbers and distant sampling of soil and plants for analysis. This resulted in 12 individually treated plots.

### 2.4. One- and Two-Year Follow-Up Studies on PA-Transfer to Acceptor-Plants on Fields Previously Used for L. squarrosa Cultivation 

These experiments were carried out on 50–400 m^2^ plots in Zappendorf (Black loess No. 2/clay soil) after harvesting *L. squarrosa* crops.

The experiments were performed growing winter wheat and spring barley as subsequent crops in the first year after cultivation. For the second year, the plots were swapped, and spring barley followed winter wheat and vice versa. Controls using the same crop varieties were grown on a nearby comparable location, with the difference being that no PA-plants (e.g., *L. squarrosa*) were grown on these plots previously. The plots were sampled three times per season: (a) soil right before the start of the cultivation, (b) soil, roots and plantlets at the two-node stage and (c) soil, roots, above ground plant parts and grain/seeds/fruits of the cultivated crop. The subsequent crops were selected according to their importance for local agriculture. A total of three seasons were covered.

In addition, a more diverse range of follow-up crops of common regional varieties from other plant families were also included in these experiments, however, not under the strict procedure of three independent replicates. These other crops included *Brassica napus*, *Pisum sativum* and *Coriandrum sativum*. These experiments were conducted nearby on similar soil and climate conditions (diluvial site, soil value index 54, Halle/Saale, Martin Luther University, Halle-Wittenberg, Germany). 

### 2.5. Investigations of Distance-Related Effects on PA-Transfer

To investigate the effects of distance on PA-transfer to non-PA-plants, field strips right next to the *L. squarrosa* cultivation were prepared. The location in 2019 was Kühnfeld (Martin Luther University, Halle-Wittenberg, Germany, soil value index 54), and in 2020 it was Zappendorf (soil value index 85, Saxony-Anhalt, Germany). These strips were planted with *Lolium multiflorum*. Plant and soil samples were taken at a distance of 50 cm, 200 cm and 400 cm away from the field of *L. squarrosa* cutivation. In addition, to monitor and minimize the PA-carry-over via soil, four Kick-Brauckmann vessels with a diameter of 22 cm and a height of 22 cm (0.038 m^2^ soil) were buried up to the rim in these strips. Two vessels each were placed at a 50 cm and two vessels at 200 cm distance to the *L. squarrosa* cultivation. All four vessels per season also contained *L. multiflorum* as plants. To illustrate this experiment, we added a picture to the Supplementary Materials (Figure S1).

### 2.6. Controls and Pot Experiments

All experiments described above were accompanied through seasons with control experiments using plots that had no history of PA-plant cultivation (in particular, *L. squarrosa*). On these control patches *Brassica napus*, *Pisum sativum*, *Coriandrum sativum*, *T. aestivum*, *H. vulgare* and *L. multiflorum* were cultivated. 

In addition to these field-controls, a one-season model experiment was conducted, using 20 Kick-Brauckmann pots (including replicates) to grow *T. aestivum* and *H. vulgare* separately, under controlled conditions. At the start of the experiment, the pots were individually filled using two kinds of soil: (a) commercial potting soil, and (b) top soil from a harvested *L. squarrosa* field. Shoot samples of both grain crops from both treatments were collected and analyzed for PAs at the two-node stage.

### 2.7. Sample Collection

In all experiments, samples of soil at the different growing stages (sowing stage, vegetation stage, two-node stage, and harvesting stage) were taken in the soil layer of the main root horizon (0–30 cm depth) using the boring stick method (8–14 impacts per variant depending on the crop acreage). All soil samples were frozen in plastic zip-lock bags and stored at −20 °C, then lyophilized and stored under dry and dark conditions until analysis. On the other hand, whole plants (including roots) at the vegetation stage (two-node stage) and crops at the stage of harvest were manually sampled using a spade (8–10 stakes) to collect about 20–50 plants per variant, depending on the species. In addition, individual plants of weeds growing in the plots were sampled as well. The plant material of the vegetation stage (two-node stage) and weeds were cleaned from adherent soil and separated into roots and shoot material. Harvest-ready plants were separated into roots, straw, and crop product (seeds, fruits); all different plant parts were collected in individual paper bags, air dried and stored at room temperature until analysis. Right before analysis all individual samples of one plot/field were well mixed resulting in an individual cross-section sample per data point, hence each value represents a biological average. 

### 2.8. Chemicals and Reagents

All chemicals used were purchased from Roth (Karlsruhe, Germany) and Sigma-Aldrich (Seelze, Germany) and of HPLC grade purity or redistilled before use. Lithium aluminum hydride solution (1 M) in THF and pyridine both in AcroSeal quality were acquired from ACROS Organics (Fair Lawn, New Jersey, USA). Strong cation exchange solid phase extraction cartridges (SCX-SPE) were obtained from Phenomenex (Aschaffenburg, Germany). Isotopically labeled internal standard (IST) 7-*O*-9-*O*-dibutyroyl-[9,9−^2^H_2_]-retronecine, was synthesized in our laboratory [28].

### 2.9. Chemical Analysis of PAs

#### 2.9.1. Sample Preparation and HPLC-ESI-MS/MS Quantification of the Total PA-Content

All analyzed samples were in homogenous form, lyophilized and homogenized in 50 mL conical centrifuge tubes using a mixer mill (MM 400; Retsch, Haan, Germany) for 2 min at 30 Hz. Three-hundred mg from each sample was soaked in a 15 mL polypropylene, conical centrifuge tube using 11 mL H_2_SO_4_ 0.05 M. Then, 40 μL of internal standard solution (2 μg/mL in methanol) was added to each tube and the tubes were thoroughly mixed and extracted overnight using a continuous tube rotator (Multi Bio RS-24, Biosan, Riga, Latvia) and the following settings: orbital: 21/01, reciprocal: 15/01, vibrio: 5/1, duration: 14 h. Sample preparation to analyze the total PA-content of the individual samples was carried out according to the methods of Cramer et al. [28] and Letsyo et al. [29]. During the course of sample preparation all 1,2-unsaturated retronecine- and heliotridine-type ester PAs/PANOs were converted into the corresponding core structures, i.e., retronecine and/or heliotridine. After derivatization, these analytes were analyzed by HPLC-ESI-MS/MS, generating a single signal which could be quantified by the use of the added isotopically labeled IST, allowing the calculation of the total content of all 1,2-unsaturated retronecine- and heliotridine-type ester PAs including all metabolites thereof, which still bear both necessary features of PA-toxicity (1,2-unsaturation and ester-PAs). Using an internal deuterated standard allowed the direct quantification across all matrices (matrix effects were covered and the IST was detected in all analyzed samples). A limit of detection (LoD) of 1 µg PA/kg and a limit of quantification (LoQ) of 5 µg PA/kg was obtained across all different matrices. A detailed description of this method and the sum parameter approach as well as the underlying calculations was added to the Supplementary Material or can be found at Cramer et al. [28]. Individual samples (positive and negative analytical results) were re-analyzed randomly, to confirm the integrity of the method. Data analysis and integration was achieved with Analyst 1.6.2 Software (Applied Biosystems MDS Sciex, Darmstadt, Germany). All analytical values are presented based on dry weight (d.w.).

#### 2.9.2. Profiling of Individual PA-Patterns by HPLC-ESI-MS/MS 

Pyrrolizidine alkaloid patterns of soil and plant materials were determined by an HPLC-ESI-MS/MS method, using an individual PA-detection approach according to the published BfR-method [30]. Extraction and profiling of the PAs in these products were conducted as a service by a commercial laboratory (QSI, Bremen, Germany; Chemisch-physikalische Analyze (#45183)) comprising 31 individual PAs and PANOs. The detailed results of this analysis can be found in the Supplementary Materials Table S2.

## 3. Results and Discussion

As outlined in the introduction, the transfer of toxic PAs from decaying PA-plant material as well as the transfer from nearby PA-plants to acceptor non-PA-plants was demonstrated in laboratory model experiments and its possible impact on food and feed safety was discussed [22,31]. In order to be able to derive realistic conclusions for a possible risk of horizontal PA-transfer from PA-plants to non-PA-plants, which might end up in food and feed, it was necessary to choose an appropriate experimental design. This means, in particular, a realistic ratio of PA-plants/PA-plant material (PA-donor) to non-PA-crop (PA-acceptor). Under given circumstances (Western agricultural economy) there should only be a very low number of PA-weeds compared to non-PA-crops. We know from several studies that these few so-called “accessory herbs” can cause PA-contamination via co-harvesting of the non-PA-crop [32,33], thus, it can most likely be ruled out that horizontal PA-transfer causes an additional risk in such a scenario. However, there is one realistic scenario which might cause problems in terms of horizontal PA-transfer that should be considered and investigated in more detail. A few PA-plant species are grown for commercial reasons. Hence, under this circumstance, there are exclusively PA-plants growing on an agricultural scale, generating potential PA-pressure directly on: (a) neighboring plants/cultivations, or indirectly via; (b) PA residues in the soil, which may impact subsequent cultivations on these plots. In addition, these PA-plant cultivations generate biomass waste that might return to fields as a form of organic fertilizer (composts, harvest residues or biogas residues) as it is promoted for circular bioeconomies. In all these scenarios there would be a potential risk that higher PA-loads might be accidently transferred to non-PA-crops, generating a significant accumulation of PAs in these acceptor-food-crops. 

Currently, there are only a few PA-plants used commercially, mainly from the Boraginaceae plant family for the production of high-quality seed oils, such as *Borago officinalis* [34], *Buglossoides arvensis* [35], and *Echium plantagineum* [36]. In our experiments, we used *Lappula squarrosa*, another PA-producing Boraginaceae species currently being investigated for its potential as an alternative source for seed oils rich in stearidonic acid [37]. In addition to these cultivated PA-plants or production residues thereof, some organic fertilizers like composts might be produced containing, e.g., press-cake from Boraginaceae seed oil production or cuttings from other PA-plants for bio-recycling. In particular, it has recently been observed that some species like *S. jacobaea* (syn.: *Jacobaea vulgaris*), *Senecio aquaticus* [38,39], or *Senecio inaequidens* (a neophyte to European areas) [40], may mass-infest pasture areas or public land (parks, greenery, nature preserves, and ancillary areas of transport routes/roadsides) [41]. Those cuttings and green wastes naturally contain a high load of toxic PAs and if used as organic fertilizers in non-PA-crops, these PAs could potentially be passed on via horizontal PA-transfer. Instead of unrealistic laboratory experiments we designed a series of field experiments to investigate the possibility and the potential risks of horizontal PA-transfer. The experiments were conducted over a period of three seasons in order to trace as precisely and comprehensively as possible the impact of “worst case” scenarios by cultivating PA-plants and recycling their biomass, e.g., as compost. The experimental design is summarized in Figure 2.

Each year, the donor-PA-plant *L. squarrosa* was cultivated on different field plots (Figure 2, Plot A) and soil and plant samples (accessory herbs) were analyzed for PA-content. Plots B and C, respectively (Figure 2), represent two fields, where *L. squarrosa* grew before. While on plot B L. squarrosa grew there the season before, the second, plot C, was free of the donor-PA-plant for one season, resulting in a two-year follow-up study on plots growing *L. squarrosa*. Here, soil and plant samples (accessory herbs) were analyzed for PA-content three times and two times during each season, respectively. Plots D and E had no history of PA-plant cultivation before and were sub-divided into small plots where different crops were grown using additional organic fertilization (Plot D, PA-plant composts; Plot E, *L. squarrosa* press-cake from seed oil production residues). Plots F and G had no history of *L. squarrosa* cultivation and were used as controls. While Plot F was a pure control plot, Plot G was a strip next to the *L. squarrosa* cultivation (starting at a distance of 50 cm) to monitor possible distance effects on neighboring non-PA-plants. All experiments were repeated using new plots where necessary (e.g., new plots for controls, composts and so on) and rotating the *L. squarrosa* follow-up crops, which means the scheme illustrated in Figure 2 was performed twice, resulting in a two-year follow-up period for plots where *L. squarrosa* was grown in the past. Furthermore, in the period from March to August 2019, pot experiments were added as additional controls using *L. squarrosa* soil from the pre-season and commercial potting soil as substrates, and *T. aestivum* and *H. vulgare* as acceptor-plants (Figure 2; Pot A and Pot F). 

### 3.1. PA-Content of Soil

The soil of all plots was sampled and was analyzed for PA-content before the cultivation started, then during the growth phase (two-node stage) as well as at the time of harvest. Since the applied analytical method was a sum parameter method, it comprised all toxicological relevant 1,2-unsaturated heliotrine- and retronecine-type PAs or even metabolized forms, which might occur due to biodegradation by soil microorganisms. Surprisingly, only some samples showed elevated PA-concentrations. Hence, the PA-positive soil results will be listed and discussed in the corresponding sections of the individual experiments below. 

### 3.2. PA-Content of Non-PA-Plants Growing on L. squarrosa Plots (Plot A)

These experiments roughly reflect the experiments conducted so far to demonstrate the phenomena of horizontal PA-transfer [21,31]. In this case, a large number of PA-plants (*L. squarrosa*) were growing next to non-PA-plants (here common endemic weeds). For this scenario, elevated levels of PAs were observed generally in roots and in the above ground plant parts of those accessory herbs. Table 1 shows the level of PAs transferred to individual non-PA-plants via horizontal transfer.

The PA-levels in these plants ranged from traces (*P. oleracea*) up to 2917.6 µg PA/kg in the roots (average: 455 µg PA/kg) and 16.6–7254.7 µg PA/kg in the shoots (average: 1995 µg PA/kg). Besides the exemption of *C. album*, the PA-levels in the shoots always exceeded the levels in the roots, here by a factor of 15 on average (shoot/root factor: ranging from 0.3 (*C. album*) to 64 (*A. patula*)). The highest levels of PAs were recorded for the shoots and in the roots of *V. arvensis* (Table 1). This corresponds well to previous findings by Nowak et al. [21] and Letsyo et al. [23], where there was also the trend of higher PA-levels in the transpiration-active organs of the acceptor-plants, like shoots and leaves. In addition, the PA-levels observed in the acceptor-plants correspond to the results reported from the laboratory-like experiments, either by the transfer of mulching/decaying PA-plants ([21]: 50–500 µg PA/kg) or by co-cultivation of acceptor-plants with *S. jacobaea* ([31]: 100–1500 µg PA/kg). However, as interesting as these observations of horizontal natural-product transfers are, in our opinion it is absolutely essential to put these numbers into perspective and in direct comparison to the corresponding donor-plants to receive a realistic impression on how relevant this transfer is in terms of possible PA-contamination. Hence, for comparison reasons, the PA-levels of *L. squarrosa* were determined as well and are given in Table 1 (last row). As a result, the PA-levels of the donor-plant are two to three times the magnitude of the levels in the acceptor-plants. Our field experiments reflected the worst-case scenario possible for a realistic agricultural practice. Accessory plant/weeds (here: non-PA-plants) growing as close as naturally possible were by far outnumbered by PA-Plants. In this setting, a transfer rate of 0–0.23% and 0–0.18% compared to the donor-PA-plant could be observed. Hence, in all other possible agricultural scenarios these ratios are exactly the other way around (a non-PA-plant is the cultivated crop and by far outnumbers some individual PA-containing weeds). As a main result of this study, a low number of PA-weeds, together with the low observed transfer rate, will not lead to detectable contaminations of final crop as a whole via horizontal PA-transfer. Instead, co-harvesting of PA-weeds together with crops can cause significant PA-levels in food and feed [13,42,43] and should be considered the major route for the possible PA-contamination.

During our standard sum parameter quantitative analytical approach the structural information of the PAs is lost. Therefore, we analyzed the individual PAs of some selected samples to obtain the PA-profile using a second analytical method, established for the simultaneous determination of 17 PAs and 14 PANOs [30]. The typical PA-pattern observed for *L. squarrosa* in this study was; lycopsamine (39.7%), lycopsamine-*N*-Ox (26.1%), intermedine-*N*-Ox (20.7%) and intermedine-*N*-Ox (13.6%) (Figure 1), all members of the 1,2-unsaturated lycopsamine-type family PAs, typical for Boraginaceae plant spp. The results of this individual PA-analysis confirmed that *C. arvensis*, *E. crus-galli*, *A. patula*, and *V. arvensis* had similar PA-patterns as observed in *L. squarrosa*, where lycopsamine ranged between 48.3–67.6%, intermedine 23.6–32.4%, lycopsamine-*N*-Ox 0–21.3%, and intermedine-*N*-Ox 0–6.9%, all *L. squarrosa* typical open-chain retronecine-monoester-type of PAs. No other PAs/PANOs e.g., from the Senecionine-type PAs (closed-ring, diester-type) were detected (Figure 1). These findings confirmed that *L. squarrosa* was always the source of the PAs found in the accessory herbs.

### 3.3. Distance Effect of PA-Transfer (Plot G)

The effect of distance on the transfer of PAs was also investigated in this study (Table 2). These plots (Plot G, Figure 2) were located directly next to *L. squarrosa* cultivations (Plot A, Figure 2). The soil PA-content before the start of the experiment was below the limit of detection. As a model, *L. multiflorum*, an additional grass variety (compared to Plots B and C), was chosen for these experiments. The PA-levels in relation to the distance from the *L. squarrosa* cultivation were measured at the full-blooming stage of the nearby *L. squarrosa* cultivation (Figure S2). Two setups were investigated and sampled: (a) regular growing grass at a certain distance; and (b) grass samples growing in an extra pot, which was buried in the soil before the start to see how a barrier in the soil (closed system in terms of soil-transfers) would affect the PA-transfer.

The results showed significantly reduced PA-transitions compared to the data obtained for the accessory herbs growing in Plot A (Figure 1). While accessory herbs in Plot A showed average values of 455 and 1995 µg PA/kg on average for roots and shoots, respectively, a distance of 50 cm to *L. squarrosa* lowered the PA-levels significantly to 19 and 335 µg PA/kg for roots and shoots, respectively. In addition, greater distances resulted in steadily decreasing PA-levels.

In all cases analyzed, the PA-profiles detected in *L. multiflorum* were matching the PA-profile of *L. squarrosa* (see Section 3.2.), with a domination of lycopsamine (66.03−77.78%) and intermidine (20.15−27.91%), which points to *L. squarrosa* as the sole PA-source. 

Surprisingly, the potted samples also still showed some low levels of PAs, mainly in the shoots. It is expected that the horizontal PA-transfer takes place via the roots; however, there seems to be the possibility that a small fraction of this transfer might occur differently. At this point in time, we assume that maybe air-borne particles (pollen or dust of the nearby *L. squarrosa* plants) or rainwater flowing on the soil surface and over the rims of the buried pots and carrying a PA-load from the neighboring *L. squarrosa* cultivation [44,45] might cause these low PA-transfers.

As a result, for agricultural practice of growing PA-plants, a distance of four meters should be an adequate isolation distance to reduce PA-contamination of neighboring cultivations and reduce PA-transfers to a minimum.

### 3.4. Crops on Fields Used for L. squarrosa Cultivation Before

In another series of experiments, we wanted to monitor the possible transfer of PAs on fields which were previously used for *L. squarrosa* cultivation, to crops that grew on these soils in the following seasons. Two different follow-up crops (two types of cereals grains) usually recommended as follow-up crops for *L. squarrosa* were used to cover for a broader picture.

#### 3.4.1. One-Year Follow-Up Studies on PA-Transfer to Acceptor-Plants on Fields Previously Used for L. *squarrosa* Cultivation (Plot B)

Several growth stages were observed. At the beginning, only soil at the stage of sowing was sampled. Later on, soil and plants at the two-node stage and just before harvest, including the crop fruits (here: cereal grains), were sampled and analyzed for total PA-content. As shown in Table 3, soil of such plots might contain low levels of PAs due to the preceding *L. squarrosa* cultivation. This PA-load can also be transferred to the next generation of crops on these plots, as it can be seen by the PA-values of the two-node stage (roots and shoots, 8.2 and 37.1 µg PA/kg, respectively), higher values were again and consistently observed in above ground parts of the plants (Table 3). 

This result for the vegetative part of the season is in accordance with Letsyo et al. [23] for *Zea mays*; there it was reported that PAs passed through the roots and accumulated at low levels in the leaves (16.3–21.1 µg/kg). Moreover, Hama et al. [44] demonstrated that during winter, soil contains lower amounts of PAs due to low temperature and the leaching of PAs into deeper soil layers out of the reach of the roots. However, at the point of harvest there were no longer PAs detected in the crops (*T. aestivum* and *H. vulgare*), suggesting that during the ripening of the grain and the die-off of the plant, the low PA-amounts of the growing phase in the green parts either vanish or get diluted below the detection limit by the increase of the above ground biomass. No PAs could be detected in the final harvest products, suggesting that fruits/grains are not a PA-sink in non-PA-plants and most importantly, no PAs would be transferred into the food chain under such circumstances.

Besides these elaborate multi-year experiments with grains (including numerous replicates of plots) individual cultivations of *C. sativum*, *P. sativum*, and *B. napus* were planted in the season right after *L. squarrosa* cultivation and monitored for PA-content. In these experiments, the soil samples at the sowing stage showed higher levels of PAs, 111–714.4 µg PA/kg (Table 4). However, while in some cases PA-soil levels were maintained at low levels throughout the season/experiment, this PA-contamination was not transferred to the later stages of the above ground plant parts nor to the harvested crops.

The additional analyzed PA-profile for the positive samples confirmed *L. squarrosa* as the origin of the PA-transfer. The detailed analytical results are summarized in the Supplementary Materials (Table S2). 

#### 3.4.2. Second-Year Follow-Up Studies on PA-Transfer to Acceptor-Plants on Fields Previously Used for *L. squarrosa* Cultivation (Plot C)

The former *L. squarrosa* plots described above were monitored for an additional season (Plot C; Figure 2), switching the follow-up crops from *T. aestivum* to *H. vulgare* and vice versa. Except for some soil and a single root sample all of these second successor crop samples were PA-negative (Table 5).

**Figure 3:**
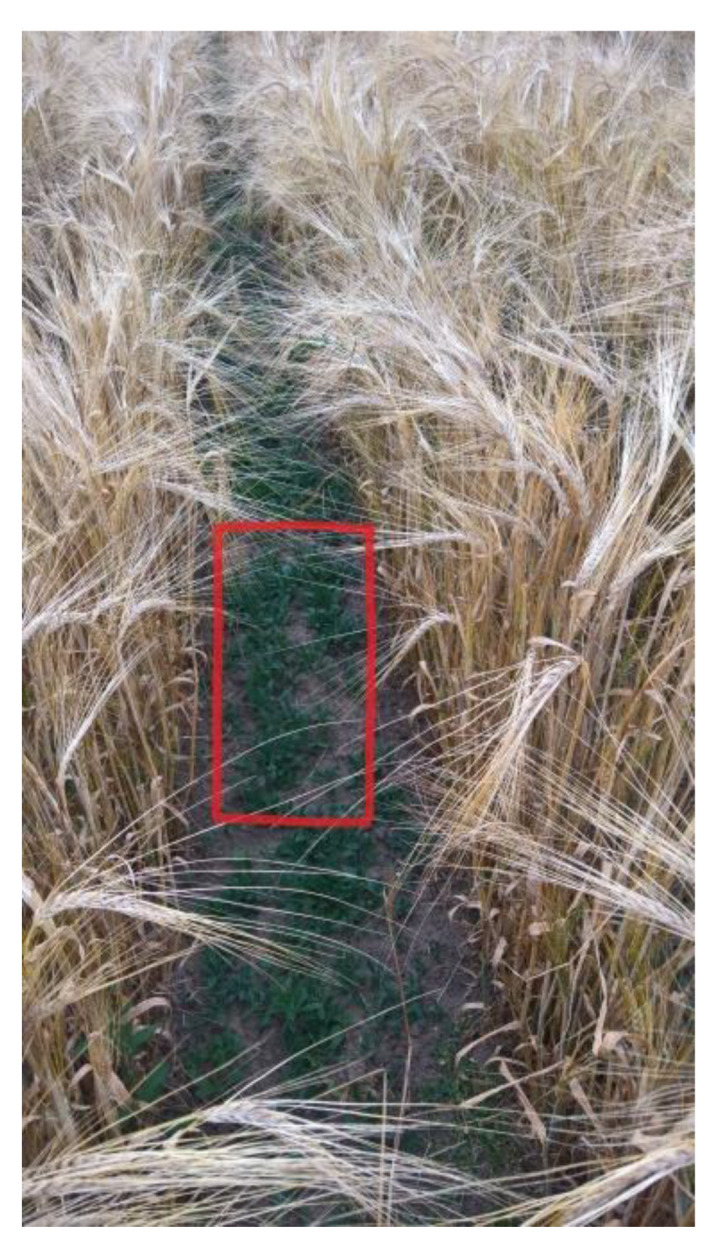
*L. squarrosa* plantlets (red box) between rows of the follow-up crop *H. vulgare*, originating from hibernated seeds of *L. squarrosa* growing on this plot two years ago (Plot C).

However, at the final stage of crop cultivation in this second season, a re-growth of new *L. squarrosa* plants appeared strictly between the rows of the sown crops (Figure 3). 

These plants originated from twice hibernated *L. squarrosa* seeds, which were still present in the soil from the *L. squarrosa* cultivation two seasons ago and which were still able to germinate. Since this somewhat resembled the situation as observed in the co-cultivation of *L. squarrosa* and accessory herbs in Plot A (Figure 2), we addressed this phenomenon in more detail. To study a possible effect of this phenomenon, three adjacent plants of *T. aestivum* and of *H. vulgare*, located right next to germinating/developing *L. squarrosa* patches, were collected and analyzed.

As shown in Table 6, roots and straw of most of these crop plants next to the *L. squarrosa* patches were PA-positive, ranging between 9.8–397.7 µg PA/kg in the roots and 13.2–108 µg PA/kg in the straw. However, there was no PA-transfer to the caryopsis of these plants. As it could be confirmed in this and other studies, the simultaneous growth of PA-plants next to non-PA-plants results in the transfer of small amounts of secondary plant metabolites. Interestingly, in this case, germinating/developing PA-plants next to “adult” acceptor-plants generally resulted in higher PA-levels in the roots instead of the shoot parts, while in the experiments before, in adult PA-donor-plants in combination with young accessory plants, this was exactly the other way around. 

The re-appearance/outgrowth of *L. squarrosa* seeds in these experiments can be attributed to the special experimental design in combination with the ecological agriculture practice applied. This led to extended row spacing allowing sunlight to reach these interspaces and promoting the development of the *L. squarrosa* plantlets. However, this does not seem a problem in general, since, due to economic reasons, the cultivation of *L. squarrosa* is intended for conventional cultivation and is very sensitive to the conventional herbicide groups and is easily outcompeted under conventional cultivation conditions with higher crop populations and denser row spacing. Hence, the possible germinating and possible carry-over of PAs seems only relevant for ecological agriculture practice and might need to be addressed there more specifically. 

The analytical determined PA-profiling confirmed that *L. squarrosa* was the source of the PA-transfer. 

### 3.5. PA-Plant Composts/L. squarrosa Press-Cake Experiments (Plots D and E)

In today’s promoted circular bioeconomies, there should be no bio-waste accumulated; instead, it should be re-utilized, and harvest residues should stay in or return to the field. This is particularly tricky if those materials contain potentially toxic compounds, in our case, the PAs. Recently, we demonstrated that a plant derived PA-load added to the composting fermentation process is dramatically reduced by more than 99.9%, while a 91 to 99% reduction was observed in bio-gas fermentation [27]. However, despite the tremendous PA-reduction through such processing, there were still some residual PA-levels in those materials ranging from 0 to 26 µg PA/kg [27].

**Figure 4:**
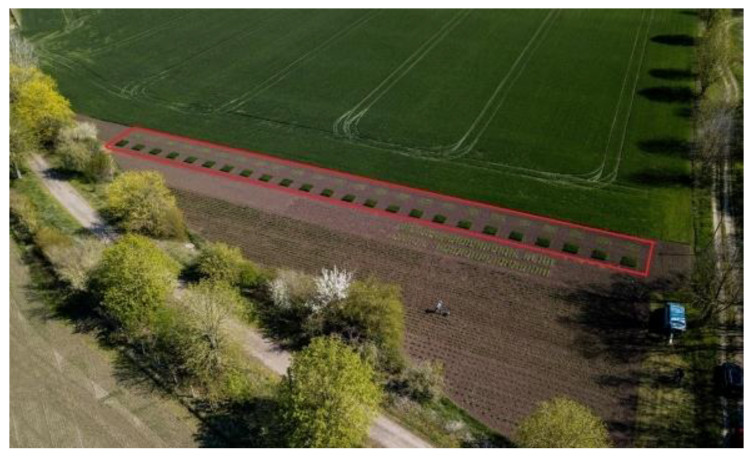
Picture showing the PA-plant composts/*L. squarrosa* press-cake experiments. *T. aestivum* (dark green) and *H. vulgare* (light green) plots are marked (red box). Each plot represents a different experimental variant or a replicate thereof.

To understand the full picture of the impact of the cultivation of PA-producing Boraginaceae species, we conducted field experiments on the effects of returned *L. squarrosa* harvest materials to the field on subsequently planted crops. In particular, we investigated different methods for improving soil quality (mainly nitrogen content) by using PA-plant composts, including *L. squarrosa* press-cake compost (residue from seed-oil production) but also used the press-cake directly (Figure 4).

In summary, all soil samples collected at sowing stage of *T. aestivum* and *H. vulgare* in 2018 and 2019 were below the limit of detection. In addition, the PA-content in soil, root, and shoot samples of the two-node vegetative stage of *T. aestivum* and of *H. vulgare* as well as all the samples at the stage of harvest, including the grains, also tested negative for PAs.

This result clearly showed that returning harvest wastes containing toxic PAs to the soil after composting or using *L. squarrosa* press-cake directly, do not lead to any risk of PA-transfers to the follow-up cultivated crops; moreover, plants, soil and farming economies benefit from this measure of returning harvest residues back to the fields. In our opinion, the so far published studies on horizontal transfer of natural products [21,31,46] do not consider common cultivation and farming conditions, instead describe a phenomenon at artificial/worst-case conditions using mulched poisonous plants (*S. jacobaea*, *C. odorata* and *C. autumnale*). It seems extremely unlikely that those conditions could be achieved under standard agricultural practice. However, it seems appropriate to reduce possible PA-loads through fermentation processes (composting, biogas fermentation) and incorporate treated plant material into the soil before the start of the next cultivation period. As demonstrated, this practice allows the safe handling and recycling of *L. squarrosa* harvest residues without any impact on follow-up cultivations.

### 3.6. Controls and Additional Pot Experiments (Plot F and Pot A and C)

As expected, in all control samples (soil, root, above ground parts and fruits) had no detectable PA-levels.

The control experiment using either fresh commercial potting soil or surface soil of a field which was used for *L. squarrosa* cultivation before, has confirmed the previous results. The analytical result showed that only those crops which grew (two-node stage) in PA-plant soil were contaminated with PAs in the shoots at levels ranging between 8.7–20.7 µg PA/kg, while the crops growing in potting soil were devoid of PAs. At the stage of harvest, on the other hand, significant levels of PAs could also be detected in the straw, but the caryopsis was PA-free. Table 7 summarizes the results obtained for PA-acceptor- plants *T. aestivum* and *H. vulgare* of pots containing PA-plant soil. 

Again, the reason for the transfer of PAs was the germinating of *L. squarrosa* plantlets in pots containing topsoil of a *L. squarrosa* field, where the seeds had fallen on the ground during harvest and germinated in the next season after hibernation. However, under this regime of forced closeness of the pot and the high abundance of *L. squarrosa* plantlets, a significant amount of PAs was found in the straw at the time of harvest (up to 2019.2 µg PA/kg). Hence, as a general preventive measure in the cultivation of PA-plants for seed oil production, the germination of remaining PA-plant seeds in the next season should be monitored and controlled, and if necessary reduced or prevented (e.g., use of herbicides). 

## 4. Conclusions

In summary, the conducted experiments help to better understand or re-evaluate two different aspects. Firstly, under certain conditions, we could reproduce the so-called “horizontal transfer of natural products”, however all our experiments did not show any marked difference to the well-known fact of the uptake of xenobiotics (organic pollutants/compounds) by plants [47]. The processes of uptake and distribution within plants are well known and mainly depend on physio/chemical characteristics of the compounds in the near environment (water, soil, air) of the acceptor-plant [47]. Whether this compound is of natural origin or synthetic seems of no relevance. Hence, the uptake is a completely neutral process and as we can extract from our data, the low transfer rates of 0.1% compared to concentrations in the “donor-plant” do not have any impact for the “acceptor”, since it results in low overall concentrations which do not benefit the acceptor (no toxic or deterrent effects can be expected which would increase the fitness). In addition, there is also no great loss for the donor, which still largely maintains its metabolite concentration (not losing fitness/protection or wasting too much energy for the production of these compounds). Therefore, there is no real “transfer”, since transfer somehow implies an intentional handover of a compound or a trait, at least from one partner. Under these circumstances of neutral and/or non-intentional (“it just happens”), using an already existing terminology like “uptake” seems to be appropriate.

Secondly, we can address the recently discussed possible negative effects of the uptake of natural products (plant toxins) and its possible impact of food and feed safety. To answer these questions, we tried to use field-trial setup spanning several seasons instead of laboratory-style experiments, using the worst-case scenario—cultivation of a PA-plant. Since the uptake only occurred at a low rate, we can define simple measures to eliminate the potential risk of PAs entering the food or feed chain. Boraginaceae seed oil plants can be safely produced by keeping distance to neighboring cultivations (e.g., using existing farm roads, ditches or the recently promoted flowering strips for biodiversity). Harvest residues can be efficiently re-used, however interposed fermenting methods (composts, biogas) are recommended, and the germinating of hibernated PA-plant seeds should be monitored and if necessary, contained appropriately. To meet or maintain quality in this specific area of PAs in food, feed or phytopharmaceuticals, the main issues for the future will still be the control and prevention of co-harvesting and processing of PA-plants together with the crop or plant of interest [3]. 

## Figures and Tables

**Table 1:** PA-content caused by horizontal PA-transfer to non-PA-accessory plants growing in *L. squarrosa* cultivations (Plot A, Figure 1).

Accessory Plant Species	Total Sum of PAs [µg PA/kg d.w.]	
	Root	Shoot
*Chenopodium album*	49.8	16.6
*Convolvulus arvensis*	512.9	4253.9
*Echinochloa crus-galli 1*	118.9	746.6
*Echinochloa crus-galli 2*	213.1	2944.2
*Echinochloa crus-galli 3*	359.2	988.5
*Echinochloa crus-galli 4*	214.4	710.1
*Echinochloa crus-galli 5*	1180.1	2184.8
*Equisetum arvense*	285.3	3691.9
*Lactuca serriola*	21.9	778.9
*Lamium purpureum*	1048.7	2975.4
*Portulaca oleracea*	trace	198.4
*Atriplex patula*	37.6	2386.6
*Stellaria media*	29.4	126.6
*Urtica dioica*	209.6	807.6
*Veronica spp.*	76.2	1850.1
*Viola arvensis*	2917.6	7254.7
*L. squarrosa* ^1^	1,251,880.0	4,053,520.0

^1^ PA-donor-plant.

**Table 2:** Effect of distance on the PA-content of *L. multiflorum* (Plot G, Figure 1) growing next to a plot of *L. squarrosa* (Plot A, Figure 1).

Distance between *L. multiflorum* and *L. squarrosa*	Total Sum of PAs [µg PA/kg d.w.]	
	Root	Shoot
50 cm without vessel	19.2	335.1
50 cm with vessel	11.9	38.1
200 cm without vessel	Trace	21.4
200 cm with vessel	Trace	8.6
400 cm without vessel	Trace	8.3

**Table 3:** PA-content of follow-up crops (*T. aestivum* and *H. vulgare*) on plots used for the cultivation of *L. squarrosa* the year before.

Sample		Total Sum of PAs [µg PA/kg d.w.]			
		*T. aestivum*		*H. vulgare*	
		2019	2020	2019	2020
sowing stage	soil	20.8	17.4	<LoD ^1^	<LoD
two-node stage	soil	<LoD	<LoD	<LoD	<LoD
	roots	8.1	10	7.4	7.3
	shoots	60.8	14.4	34.4	38.7
time of harvest	soil	<LoD	<LoD	<LoD	<LoD
	roots	<LoD	<LoD	<LoD	<LoD
	straw	<LoD	<LoD	<LoD	<LoD
	caryopses	<LoD	<LoD	<LoD	<LoD

^1^ Limit of detection.

**Table 4:** PA-content of various follow-up crops on plots used for the cultivation of *L. squarrosa* the year before.

		Total Sum of PAs [µg PA/kg d.w.]		
		*C. sativum*	*P. sativum*	*B. napus*
sowing stage	soil	714.4	111.0	332.7
two-node stage	soil	27.4	11.3	<LoD ^1^
	roots	<LoD	12.0	<LoD
	shoots	<LoD	<LoD	<LoD
time of harvest	soil	13.4	8.3	8.7
	roots	<LoD	8.3	<LoD
	straw	<LoD	<LoD	<LoD
	caryopses	<LoD	<LoD	<LoD

^1^ Limit of detection.

**Table 5:** PA-content of follow-up crops (*T. aestivum* and *H. vulgare*) on plots used for the cultivation of *L. squarrosa* two years before.

Sample		Total Sum of PAs [µg PA/kg d.w.]			
		*T. aestivum*		*H. vulgare*	
		2019	2020	2019	2020
sowing stage	soil	<LoD ^1^	15.4	<LoD	<LoD
two-node stage	soil	<LoD	<LoD	<LoD	<LoD
	root	<LoD	<LoD	8.1	32.6
	shoot	61.7	<LoD	<LoD	<LoD
time of harvest	soil	<LoD	15.7	<LoD	25.9
	roots	<LoD	<LoD	<LoD	44.8
	straw	<LoD	<LoD	<LoD	<LoD
	caryopses	<LoD	<LoD	<LoD	<LoD

^1^ Limit of detection.

**Table 6:** PA-content caused by horizontal PA-transfer to non-PA-accessory plants originating from re-germination of hibernated *L. squarrosa* seeds maintained in the soil after two years.

	Total Sum of PAs [µg PA/kg d.w.]					
	*T. aestivum*			*H. vulgare*		
	Plant 1	Plant 2	Plant 3	Plant 1	Plant 2	Plant 3
root	70.6	<LoD ^1^	20.9	<LoD	397.7	9.8
straw	66.1	<LoD	108	13.2	41.8	<LoD
caryopses	<LoD	<LoD	<LoD	<LoD	<LoD	<LoD

^1^ Limit of detection.

**Table 7:** PA-content of *T. aestivum* and *H. vulgare* growing in pots with surface soil from *L. squarrosa* fields of the previous year.

		Total Sum of PAs [µg PA/kg d.w.]	
		*T. aestivum*	*H. vulgare*
sowing stage	soil	<LoD ^1^	<LoD
two-node stage	soil	<LoD	<LoD
	roots	<LoD	<LoD
	shoots	20.7	8.7
time of harvest	soil	79.1	179.5
	roots	71.5	84.2
	straw	132.4	2019.2
	caryopses	<LoD	<LoD

^1^ Limit of detection.

## Supplementary Materials

The following are available online at https://www.mdpi.com/article/10.3390/foods10081827/s1. Figure S1. Setting up of compost-bins; A: Control-Compost, B: *S. jacobaea*-Compost, C: *S. jacobaea* and *L. squarrosa*-Compost, D: *L. squarrosa*-Compost. 1-compost starter, 2-*S. jacobaea* material, 3-bio starter, 4-compost stock, 5-*L. squarrosa* press cake. Table S1 Calculated amounts of compost/press-cakes for each plot to meet nutritional requirements. Table S2 The detailed analysis results of extraction and profiling of the PAs of plant samples as Chemisch-physikalische Analyse (#45183) comprising 31 individual PAs and PANOs by QSI (Bremen, Germany).Figure S2. Picture to illustrate investigations of distance-related effects on PA-transfer. Green: *L. squarrosa* field; Red: Excavated Kick-Brauckmann vessel (50 cm) next to its hole; Black: field strip of *L. multiflorum*; Blue: Still buried Kick-Brauckmann vessel (200 cm) including *L. multiflorum*.

## References

[1] Malysheva S.V., Mulder P.P.J., Masquelier J. (2020). Development and Validation of a UHPLC-ESI-MS/MS Method for Quantification of Oleandrin and Other Cardiac Glycosides and Evaluation of Their Levels in Herbs and Spices from the Belgian Market. Toxins.

[2] Izcara S., Casado N., Morante-Zarcero S., Sierra I. (2020). A Miniaturized QuEChERS Method Combined with Ultrahigh Liquid Chromatography Coupled to Tandem Mass Spectrometry for the Analysis of Pyrrolizidine Alkaloids in Oregano Samples. Foods.

[3] Steinhoff B. (2021). Pyrrolizidine Alkaloid Contamination in Medicinal Plants: Regulatory Requirements and Their Impact on Production and Quality Control of Herbal Medicinal Products. Planta Med..

[4] Hartmann T., Witte L., Pelletier S.W. (1995). Chemistry, biology and chemoecology of the pyrrolizidine alkaloids. Alkaloids: Chemical and Biological Perspectives.

[5] WHO (1988). Pyrrolizidine alkaloids. Environmental Health Criteria Geneva: World Health Organ. http://www.inchem.org/documents/ehc/ehc/ehc080.htm.

[6] Schrenk D., Gao L., Lin G., Mahony C., Mulder P.P.J., Peijnenburg A., Pfuhler S., Rietjens I.M.C.M., Rutz L., Steinhoff B. (2020). Pyrrolizidine alkaloids in food and phytomedicine: Occurrence, exposure, toxicity, mechanisms, and risk assessment—A review. Food Chem. Toxicol..

[7] Xu J., Wang W., Yang X., Xiong A., Yang L., Wang Z. (2019). Pyrrolizidine alkaloids: An update on their metabolism and hepatotoxicity mechanism. Liver Res..

[8] Fu P., Xia Q., Lin G., Chou M. (2004). Pyrrolizidine alkaloids—Genotoxicity, metabolism enzymes, metabolic activation, and mechanisms. Drug Metab. Rev..

[9] Moreira R., Pereira D.M., Valentão P., Andrade P.B. (2018). Pyrrolizidine Alkaloids: Chemistry, Pharmacology, Toxicology and Food Safety. Int. J. Mol. Sci..

[10] (EFSA) European Food Safety Authority (2011). Scientific opinion on pyrrolizidine alkaloids in food and feed. EFSA panel on contaminants in the food chain (CONTAM). Scientific opinion on pyrrolizidine alkaloids in food and feed. EFSA J..

[11] (EFSA) European Food Safety Authority Risks for Human Health Related to the Presence of Pyrrolizidine Alkaloids in Honey, Tea, Herbal Infusions and Food Supplements. EFSA Panel on Contaminants in the Food Chain (CONTAM) 2017. https://efsa.onlinelibrary.wiley.com/doi/epdf/10.2903/j.efsa.2017.4908.

[12] Mulder P.P.J., López Sánchez P.L., These A., Preiss-Weigert A., Castellari M. (2015). Occurrence of Pyrrolizidine Alkaloids in food. EFSA Support. Publ..

[13] Kakar F., Akbarian Z., Leslie T., Mustafa M., Watson J., Egmond H., Omar M., Mofleh J. (2010). An Outbreak of Hepatic Veno-Occlusive Disease in Western Afghanistan Associated with Exposure to Wheat Flour Contaminated with Pyrrolizidine Alkaloids. J. Toxicol..

[14] Kempf M., Heil S., Haßlauer I., Schmidt L., von der Ohe K., Theuring C., Reinhard A., Schreier P., Beuerle T. (2010). Pyrrolizidine alkaloids in pollen and pollen products. Mol. Nutr. Food Res..

[15] Matteo A., Lucchetti M.A., Glauser G., Kilchenmann V., Dübecke A., Beckh G., Praz C., Kast C. (2020). Pyrrolizidine Alkaloids from *Echium vulgare* in Honey Originate Primarily from Floral Nectar. J. Agric. Food Chem..

[16] Selmar D., Radwan A., Nowak M. (2015). Horizontal Natural Product Transfer: A so far Unconsidered Source of Contamination of Plant-Derived Commodities. Environ. Anal. Toxicol..

[17] Pullagurala V.L.R., Rawat S., Adisa I.O., Hernandez-Viezcas J.A., Peralta-Videa J.R., Gardea-Torresdey J.L. (2018). Plant uptake and translocation of contaminants of emerging concern in soil. Sci. Total Environ..

[18] Eggen T., Heimstad E.S., Stuanes A.O., Norli H.R. (2013). Uptake and translocation of organophosphates and other emerging contaminants in food and forage crops. Environ. Sci. Pollut. Res..

[19] Fismes J., Perrin-Ganier C., Empereur-Bissonnet P., Morel J.L. (2002). Soil-to-Root Transfer and Translocation of Polycyclic Aromatic Hydrocarbons by Vegetables Grown on Industrial Contaminated Soils. J. Environ. Qual..

[20] Edgar J.A., Molyneux R.J., Colegate S.M. (2020). Linking Dietary Exposure to 1,2-Dehydropyrrolizidine Alkaloids with Cancers and Chemotherapy-Induced Pulmonary and Hepatic Veno-Occlusive Diseases. J. Agric. Food Chem..

[21] Nowak M., Wittke C., Lederer I., Klier B., Kleinwächter M., Selmar D. (2016). Interspecific transfer of pyrrolizidine alkaloids: A so far unconsidered source of contaminations of phytopharmaceuticals and plant derived commodities. Food Chem..

[22] Yahyazadeh M., Nowak M., Kima H., Selmar D. (2017). Horizontal natural product transfer: A potential source of alkaloidal contaminants in phytopharmaceuticals. Phytomedicine.

[23] Letsyo E., Adams Z., Dzikunoo J., Asante-Donyinah D. (2020). Uptake and accumulation of pyrrolizidine alkaloids in the tissues of maize (*Zea mays* L.) plants from the soil of a 4-year-old Chromolaena odorata dominated fallow farmland. Chemosphere.

[24] Wagner F., Prediger G., Tiggemann B., Schmidt I. (2007). Der Feldversuch-Durchführung und Technik.

[25] Schliephake W., Garz J., Schmidt L. (1999). Exkursionsführer zu den Dauerversuchen auf dem Julius-Kühn-Versuchsfeld Halle.

[26] Wikipedia. https://de.wikipedia.org/wiki/Ackerzahl.

[27] Chmit M.S., Müller J., Wiedow D., Horn G., Beuerle T. (2021). Biodegradation and utilization of crop residues contaminated with poisonous pyrrolizidine alkaloids. J. Environ. Manag..

[28] Cramer L., Schiebel H., Ernst L., Beuerle T. (2013). Pyrrolizidine alkaloids in the food chain: Development, validation, and application of a new HPLC-ESI-MS/MS sum parameter method. J. Agric. Food Chem..

[29] Letsyo E., Jerz G., Winterhalter P., Lindigkeit R., Beuerle T. (2017). Incidence of pyrrolizidine alkaloids in herbal medicines from German retail markets: Risk assessments and implications to consumers. Phytother. Res..

[30] BfR (2014). Bestimmung von PA in Pflanzenmaterial Mittels SPE-LC-MS/MS. BfR-PA-Tee-2.0. https://www.bfr.bund.de/cm/343/bestimmung-von-pyrrolizidinalkaloiden.pdf.

[31] Selmar D., Wittke C., Beck-von Wolffersdorff I., Klier B., Lewerenz L., Kleinwächter M., Nowak M. (2019). Transfer of pyrrolizidine alkaloids between living plants: A disregarded source of contaminations. Environ. Pollut..

[32] Wiesner J. (2021). Regulatory Perspectives of Pyrrolizidine Alkaloid Contamination in Herbal Medicinal Products. Planta Med..

[33] Van Wyk B., Stander M., Long H. (2017). Senecio angustifolius as the major source of pyrrolizidine alkaloid contamination of rooibos tea (*Aspalathus linearis*). S. Afr. J. Bot..

[34] Navarro-Herrera D., Aranaz P., Eder-Azanza L., Zabala M., Romo-Hualde A., Hurtado C., Calavia D., Lopez-Yoldi M., Martinez J.A., Gonzalez-Navarro C.J. (2018). *Borago officinalis* seed oil (BSO), a natural source of omega-6 fatty acids, attenuates fat accumulation by activating peroxisomal beta-oxidation both in *C*. *elegans* and in diet-induced obese rats. Food Funct..

[35] Lefort N., LeBlanc R., Giroux M.A., Surette M.E. (2016). Consumption of *Buglossoides arvensis* seed oil is safe and increases tissue long-chain n-3 fatty acid content more than flax seed oil-results of a phase I randomised clinical trial. J. Nutr. Sci..

[36] Kitessa S.M., Nichols P.D., Abeywardena M., Preedy V.R., Watson R.R., Patel V.B. (2011). Purple Viper’s Bugloss (*Echium plantagineum*) Seed Oil in Human Health. Nuts and Seeds in Health and Disease Prevention.

[37] Cramer L., Fleck G., Horn G., Beuerle T. (2014). Process Development of *Lappula squarrosa* Oil Refinement: Monitoring of Pyrrolizidine Alkaloids in Boraginaceae Seed Oils. J. Am. Oil Chem. Soc..

[38] Chizzola R., Bassler-Binder G., Karrer G., Kriechbaum M. (2019). Pyrrolizidine alkaloid production of *Jacobaea aquatica* and contamination of forage in meadows of Northern Austria. Grass Forage Sci..

[39] Zacharias P. (2018). Offene Verwaltungsdaten zur Analyse des Befallspotenzials von Grünlandbeständen mit Schadpflanzen am Beispiel von Kreuzkräutern. GIS-Z Geoinformatik.

[40] Eller A., Chizzola R. (2016). Seas onal va riability in pyrr olizidine alkaloids in *Senecio inaequidens* from the va l venosta (northern Italy). Plant Biosyst..

[41] Lattrell B. PA-haltige Pflanzen auf nicht landwirtschaftlich genutzten Flächen: BFR-Forum Verbraucherschutz “Pyrrolizidinalkaloide -Herausforderungen an Landwirtschaft und Verbraucherschutz”. Bundesinstitut für Risikobewertung 2015. https://www.bfr.bund.de/cm/343/pa-haltige-pflanzen-auf-nicht-landwirtschaftlich-genutzten-flaechen.pdf.

[42] Azadbakht M., Talavaki M. (2003). Qualitative and Quantitative Determination of Pyrrolizidine Alkaloids of Wheat and Flour Contaminated with Senecio in Mazandaran Province Farms. Iran. J. Pharm. Sci..

[43] Kaltner F., Kukula V., Gottschalk C. (2020). Screening of food supplements for toxic pyrrolizidine alkaloids. J. Consum. Prot. Food Saf..

[44] Hama J., Strobel B. (2021). Occurrence of pyrrolizidine alkaloids in ragwort plants, soils and surface waters at the field scale in grassland. Sci. Total Environ..

[45] Kisielius V., Hama J.R., Skrbic N., Hansen H.C.B., Strobel B.W., Rasmussen L.H. (2020). The invasive butterbur contaminates stream and seepage water in groundwater wells with toxic pyrrolizidine alkaloids. Sci. Rep..

[46] Grant K., Jilg A., Messner J., Elsäßer M. (2020). Herbstzeitlose (*Colchicum autumnale*) in Mulchschicht führt zu leichter Kontamination mit Colchizin eines Pflanzenbestandes im Folgeaufwuchs. Deutsche Arbeitsbesprechung über Fragen der Unkrautbiologie und-bekämpfung, 3.-März in Braunschweig. Open J. Syst..

[47] Verkleij J.S.C., Golan-Goldhirsh A., Antosiewisz D.M., Schwitzguébel J.P., Schröder P. (2009). Dualities in plant tolerance to pollutants and their uptake and translocation to the upper plant parts. Environ. Exp. Bot..

