# Supplementary material for: Pyrrolizidine Alkaloids in the Food Chain: Is Horizontal Transfer of Natural Products of Relevance?
Source: Foods. 2021 Aug 7;10(8):1827. doi: 10.3390/foods10081827 (PMC8392022; doi:10.3390/foods10081827)
Supplement: Supplementary file 1 [file foods-10-01827-s001.zip › NEW foods-1332154-supplementary materials V210818.pdf]

Supplement:

Pyrrolizidine Alkaloids in the Food Chain: Is Horizontal Transfer of Natural Products of Relevance?

Mohammad Said Chmit[a], Gerd Horn[b], Arne Dübecke[c], Till Beuerle[a,*]

[a] Technical University of Braunschweig, Institute of Pharmaceutical Biology, Mendelssohnstr. 1, 38106 Braunschweig, Germany.
[b] Exsemine GmbH, Am Wehr 4, 06198 Salzatal, Germany.
[c] Quality Services International GmbH, Flughafendamm 9a, 28199 Bremen, Germany.
* Corresponding author

Contents

- Set-up of compost bins p. 3
- Analysis results of soil nutrients by ÖHMI (Magdeburg, Germany); Control-compost 2018, *Senecio*-compost 2018, *Senecio*/*Lappula*-compost 2018, *Lappula*-compost 2018, *Lappula squarrosa*-press cake, Control-compost 2019, *Senecio*-compost 2019, *Senecio*/*Lappula*-compost 2019, and *Lappula*-compost 2019 respectively. pp. 4-12
- Calculated amounts of compost/press-cakes added to each plot to meet the nutrient requirements p. 13
- Parameters of HPLC-ESI-MS/MS sum parameter analysis p. 14
- Summarized analytical results comprising 31 individual PAs and PANOs conducted by QSI (#45183) p. 15
- Picture to illustrate Investigations of distance-related effects on PA-transfer p. 16

Composition of individual composts

Control-compost

The control- compost was build up according to the following ratios: 88% compost stock, 9.5% compost starter and 2.5% bio starter.

Senecio-compost

S. jacobaea-compost contains the following components:
56% compost stock, 9.5% compost starter, 2.5% bio starter and 32% *S. jacobaea* material.

Senecio/Lappula-compost

To build up *S. jacobaea* - and *L. squarrosa*- Compost the following components were used:
64% compost stock, 9.5% compost starter, 2.5% bio starter, 15% *S. jacobaea* material and 9% *L. squarrosa* press cake powder.

Lappula-compost

L. squarrosa-compost was created from: 74% compost stock, 9.5% compost starter, 2.5% bio starter and 14% *L. squarrosa* press cake powder.

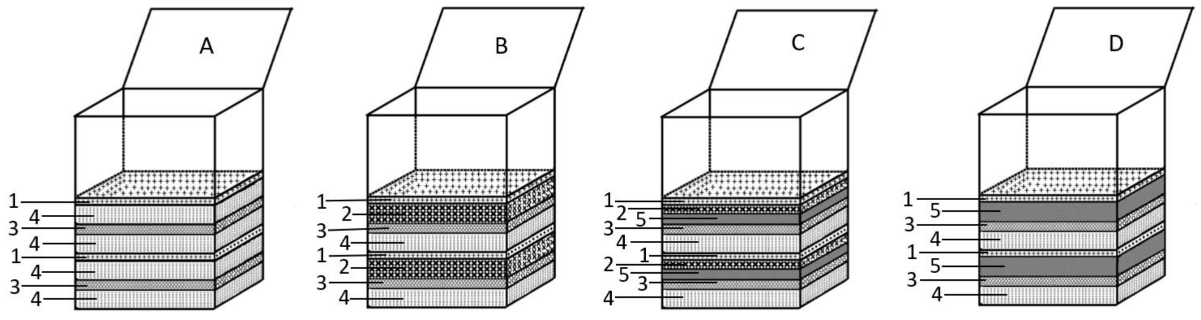

Fig. S1. Setting up of compost-bins; A: Control- Compost, B: *S. jacobaea*- Compost, C: *S. jacobaea* and *L. squarrosa*-Compost, D: *L. squarrosa*- Compost. 1- compost starter, 2- *S. jacobaea* material, 3- bio starter, 4- compost stock, 5- *L. squarrosa* press cake.

Magdeburg,
09.10.2018

Prüfbericht

Prüfbericht-Nr.:
A 18.2632

Auftraggeber: EXSEMINE GmbH
Am Wehr 4
06198 Salzatal OT Zappendorf

Email: g.horn@exsemine.de

Auftragnehmer/
Ort der Labortätigkeit: ÖHMI Analytik GmbH
Berliner Chaussee 66
39114 Magdeburg

Probenahme: erfolgte durch Auftraggeber

Eingangsparameter: 02.10.2018 / 14:40 Uhr / 15,5 °C

Lagerung vor Untersuchung: Kühllagerung bei max. 5,0 °C

Untersuchungsbeginn: 02.10.2018 / 14:55 Uhr

Untersuchungsende: 09.10.2018

Probenbezeichnung: Laut Kundeninformation:
Kompost 1C
=A 18.2632

Kunden-Proben-Nr.:			Kompost 1C
ÖHMI-Proben-Nr.: A 18.2632/1			
Kennzahl	Dimension	Methode	A 18.2632/1
Trocken-rückstand	%	DIN EN 12880 Stand: 02-2001	48,7
Gesamt-N	% OS	Methodenbuch VDLUFA + Berechnung	0,54
**CAL-Extrakt**	kg/t OS	Methodenbuch VDLUFA	
P	kg/t OS	DIN EN ISO 11885 Stand:09-2009	0,69
K	kg/t OS	DIN EN ISO 11885 Stand: 09-2009	1,04
**$CaCl_2$-Extrakt**			
Ammonium-N	kg/t OS	E DIN ISO 14256 Stand:03-2003	0,0054

OS = Originalsubstanz

Bianca Denecke
Abteilungsleiterin Chemie

Die Prüfergebnisse beziehen sich ausschließlich auf das untersuchte Prüfmaterial. Sollte die Probenahme nicht durch die ÖHMI Analytik GmbH erfolgt sein, beziehen sich die Ergebnisse auf die Probe wie erhalten. Ohne schriftliche Genehmigung des Prüflaboratoriums darf der Prüfbericht nicht auszugsweise vervielfältigt oder veröffentlicht werden. Es gelten unsere Allgemeinen Geschäftsbedingungen. Mit Auftragserteilung gelten die Allgemeinen Geschäftsbedingungen als anerkannt.

Magdeburg,
09.10.2018

Prüfbericht

Prüfbericht-Nr.:
A 18.2633

Auftraggeber: EXSEMINE GmbH
Am Wehr 4
06198 Salzatal OT Zappendorf

Email: g.horn@exsemine.de

Auftragnehmer/ Ort der Labortätigkeit: ÖHMI Analytik GmbH
Berliner Chaussee 66
39114 Magdeburg

Probenahme: erfolgte durch Auftraggeber

Eingangsparameter: 02.10.2018 / 14:40 Uhr / 15,5 °C

Lagerung vor Untersuchung: Kühllagerung bei max. 5,0 °C

Untersuchungsbeginn: 02.10.2018 / 14:55 Uhr

Untersuchungsende: 09.10.2018

Probenbezeichnung: Laut Kundeninformation:
Kompost 2S
=A 18.2633

Kunden-Proben-Nr.:			Kompost 2 S
ÖHMI-Proben-Nr.: A 18.2633/1			
Kennzahl	Dimension	Methode	A 18.2633/1
Trocken-rückstand	%	DIN EN 12880<br>Stand: 02-2001	49,4
Gesamt-N	% OS	Methodenbuch VDLUFA + Berechnung	0,68
**CAL-Extrakt**	kg/t OS	Methodenbuch VDLUFA	
P	kg/t OS	DIN EN ISO 11885<br>Stand:09-2009	1,21
K	kg/t OS	DIN EN ISO 11885<br>Stand: 09-2009	2,15
**$CaCl_2$-Extrakt**			
Ammonium-N	kg/t OS	E DIN ISO 14256<br>Stand:03-2003	0,015

OS = Originalsubstanz

Bianca Denecke
Abteilungsleiterin Chemie

Die Prüfergebnisse beziehen sich ausschließlich auf das untersuchte Prüfmaterial. Sollte die Probenahme nicht durch die ÖHMI Analytik GmbH erfolgt sein, beziehen sich die Ergebnisse auf die Probe wie erhalten. Ohne schriftliche Genehmigung des Prüflaboratoriums darf der Prüfbericht nicht auszugsweise vervielfältigt oder veröffentlicht werden. Es gelten unsere Allgemeinen Geschäftsbedingungen. Mit Auftragserteilung gelten die Allgemeinen Geschäftsbedingungen als anerkannt.

Magdeburg,
09.10.2018

Prüfbericht

Prüfbericht-Nr.:
A 18.2634

Auftraggeber: EXSEMINE GmbH
Am Wehr 4
06198 Salzatal OT Zappendorf

Email: g.horn@exsemine.de

Auftragnehmer/
Ort der Labortätigkeit: ÖHMI Analytik GmbH
Berliner Chaussee 66
39114 Magdeburg

Probenahme: erfolgte durch Auftraggeber

Eingangsparameter: 02.10.2018 / 14:40 Uhr / 15,5 °C

Lagerung vor Untersuchung: Kühllagerung bei max. 5,0 °C

Untersuchungsbeginn: 02.10.2018 / 14:55 Uhr

Untersuchungsende: 09.10.2018

Probenbezeichnung: Laut Kundeninformation:
Kompost 3 SL
=A 18.2634

Kunden-Proben-Nr.:			Kompost 3 SL
ÖHMI-Proben-Nr.:	A 18.2634/1		
Kennzahl	Dimension	Methode	A 18.2634/1
Trocken-rückstand	%	DIN EN 12880 Stand: 02-2001	46,1
Gesamt-N	% OS	Methodenbuch VDLUFA + Berechnung	0,66
CAL-Extrakt	kg/t OS	Methodenbuch VDLUFA	
P	kg/t OS	DIN EN ISO 11885 Stand:09-2009	1,58
K	kg/t OS	DIN EN ISO 11885 Stand: 09-2009	1,87
$CaCl_2$-Extrakt			
Ammonium-N	kg/t OS	E DIN ISO 14256 Stand:03-2003	0,013

OS = Originalsubstanz

Bianca Denecke
Abteilungsleiterin Chemie

Die Prüfergebnisse beziehen sich ausschließlich auf das untersuchte Prüfmaterial. Sollte die Probenahme nicht durch die ÖHMI Analytik GmbH erfolgt sein, beziehen sich die Ergebnisse auf die Probe wie erhalten. Ohne schriftliche Genehmigung des Prüflaboratoriums darf der Prüfbericht nicht auszugsweise vervielfältigt oder veröffentlicht werden. Es gelten unsere Allgemeinen Geschäftsbedingungen. Mit Auftragserteilung gelten die Allgemeinen Geschäftsbedingungen als anerkannt.

Magdeburg,
09.10.2018

Prüfbericht-Nr.:
A 18.2635

Prüfbericht

Auftraggeber: EXSEMINE GmbH
Am Wehr 4
06198 Salzatal OT Zappendorf

Email: g.horn@exsemine.de

Auftragnehmer/
Ort der Labortätigkeit: ÖHMI Analytik GmbH
Berliner Chaussee 66
39114 Magdeburg

Probenahme: erfolgte durch Auftraggeber

Eingangsparameter: 02.10.2018 / 14:40 Uhr / 15,5 °C

Lagerung vor Untersuchung: Kühllagerung bei max. 5,0 °C

Untersuchungsbeginn: 02.10.2018 / 14:55 Uhr

Untersuchungsende: 09.10.2018

Probenbezeichnung: Laut Kundeninformation:
Kompost 4 L
= A 18.2635

Kunden-Proben-Nr.:			Kompost 4 L
ÖHMI-Proben-Nr.:	A 18.2635/1		
Kennzahl	Dimension	Methode	A 18.2635/1
Trocken-rückstand	%	DIN EN 12880 Stand: 02-2001	47,2
Gesamt-N	% OS	Methodenbuch VDLUFA + Berechnung	0,69
**CAL-Extrakt**	kg/t OS	Methodenbuch VDLUFA	
P	kg/t OS	DIN EN ISO 11885 Stand:09-2009	1,78
K	kg/t OS	DIN EN ISO 11885 Stand: 09-2009	2,15
**$CaCl_2$-Extrakt**			
Ammonium-N	kg/t OS	E DIN ISO 14256 Stand:03-2003	0,10

OS = Originalsubstanz

Bianca Denecke
Abteilungsleiterin Chemie

Die Prüfergebnisse beziehen sich ausschließlich auf das untersuchte Prüfmaterial. Sollte die Probenahme nicht durch die ÖHMI Analytik GmbH erfolgt sein, beziehen sich die Ergebnisse auf die Probe wie erhalten. Ohne schriftliche Genehmigung des Prüflaboratoriums darf der Prüfbericht nicht auszugsweise vervielfältigt oder veröffentlicht werden. Es gelten unsere Allgemeinen Geschäftsbedingungen. Mit Auftragserteilung gelten die Allgemeinen Geschäftsbedingungen als anerkannt.

Magdeburg,
09.10.2018

Prüfbericht

Prüfbericht-Nr.:
A 18.2636

Auftraggeber: EXSEMINE GmbH
Am Wehr 4
06198 Salzatal OT Zappendorf

Email: g.horn@exsemine.de

**Auftragnehmer/
Ort der Labortätigkeit:** ÖHMI Analytik GmbH
Berliner Chaussee 66
39114 Magdeburg

Probenahme: erfolgte durch Auftraggeber

Eingangsparameter: 02.10.2018 / 14:40 Uhr / 15,5 °C

Lagerung vor Untersuchung: Kühllagerung bei max. 5,0 °C

Untersuchungsbeginn: 02.10.2018 / 14:55 Uhr

Untersuchungsende: 09.10.2018

Probenbezeichnung: Presskuchen Lappula squarrosa Press 6-7/16,
gemahlen 18.4.18
=A 18.2636

Kunden-Proben-Nr.:			Presskuchen Lappula squarrosa Press 6-7/16, gemahlen 18.4.18
ÖHMI-Proben-Nr.:	A 18.2636/1		
Kennzahl	Dimension	Methode	A 18.2636/1
Trocken-rückstand	%	DIN EN 12880 Stand: 02-2001	88,4
Gesamt-N	% OS	Methodenbuch VDLUFA + Berechnung	3,0
**CAL-Extrakt**	kg/t OS	Methodenbuch VDLUFA	
P	kg/t OS	DIN EN ISO 11885 Stand:09-2009	8,27
K	kg/t OS	DIN EN ISO 11885 Stand: 09-2009	2,83
**$CaCl_2$-Extrakt**			
Ammonium-N	kg/t OS	E DIN ISO 14256 Stand:03-2003	< 0,001

OS = Originalsubstanz

Bianca Denecke
Abteilungsleiterin Chemie

Die Prüfergebnisse beziehen sich ausschließlich auf das untersuchte Prüfmaterial. Sollte die Probenahme nicht durch die ÖHMI Analytik GmbH erfolgt sein, beziehen sich die Ergebnisse auf die Probe wie erhalten. Ohne schriftliche Genehmigung des Prüflaboratoriums darf der Prüfbericht nicht auszugsweise vervielfältigt oder veröffentlicht werden. Es gelten unsere Allgemeinen Geschäftsbedingungen. Mit Auftragserteilung gelten die Allgemeinen Geschäftsbedingungen als anerkannt.

ÖHMI

Magdeburg,
16.10.2019

Prüfbericht

Prüfbericht-Nr.:
A19.2881

Auftraggeber: EXSEMINE GmbH
Am Wehr 4
06198 Salzatal OT Zappendorf

Email: g.horn@exsemine.de

Auftragnehmer / Ort der Labortätigkeit: ÖHMI Analytik GmbH
Berliner Chaussee 66
39114 Magdeburg

Probenahme: erfolgte durch Auftraggeber

Eingangsparameter: 14.10.2019 / 13:30 Uhr / 16,4 °C

Lagerung vor Untersuchung: Kühllagerung bei max. 5,0 °C

Untersuchungsbeginn: 14.10.2019 / 13:45 Uhr

Untersuchungsende: 16.10.2019

Probenbezeichnung: lt. Kundeninformation:
Kompost 1C
Kontrolle ohne PAs
= A19.2881

Kunden-Proben-Nr.:			Kompost 1C<br>Kontrolle ohne PAs
ÖHMI-Proben-Nr.:			
Kennzahl	Dimension	Methode	A19.2881
Trocken-rückstand	%	DIN EN 12880<br>Stand: 02-2001	59,4
Gesamt-Stickstoff	% OS	Methodenbuch VDLUFA	0,62
CAL-Extrakt		Methodenbuch VDLUFA	
Phosphor	kg/t OS	DIN EN ISO 11885<br>Stand: 09-2009	0,718
Kalium	kg/t OS	DIN EN ISO 11885<br>Stand: 09-2009	0,494
$CaCl_2$-Extrakt		Methodenbuch VDLUFA	
Ammonium-Stickstoff	kg/t OS	E DIN ISO 14256<br>Stand: 03-2003	0,0013

OS = Originalsubstanz

Bianca Denecke
Abteilungsleiterin Chemie

Die Prüfergebnisse beziehen sich ausschließlich auf das untersuchte Prüfmaterial. Sollte die Probenahme nicht durch die ÖHMI Analytik GmbH erfolgt sein, beziehen sich die Ergebnisse auf die Probe wie erhalten. Ohne schriftliche Genehmigung des Prüflaboratoriums darf der Prüfbericht nicht auszugsweise vervielfältigt oder veröffentlicht werden. Es gelten unsere Allgemeinen Geschäftsbedingungen. Mit Auftragserteilung gelten die Allgemeinen Geschäftsbedingungen als anerkannt.

ÖHMI

Magdeburg,
16.10.2019

Prüfbericht

Prüfbericht-Nr.:
A19.2882

Auftraggeber: EXSEMINE GmbH
Am Wehr 4
06198 Salzatal OT Zappendorf

Email: g.horn@exsemine.de

Auftragnehmer / Ort der Labortätigkeit: ÖHMI Analytik GmbH
Berliner Chaussee 66
39114 Magdeburg

Probenahme: erfolgte durch Auftraggeber

Eingangsparameter: 14.10.2019 / 13:30 Uhr / 16,4 °C

Lagerung vor Untersuchung: Kühllagerung bei max. 5,0 °C

Untersuchungsbeginn: 14.10.2019 / 13:45 Uhr

Untersuchungsende: 16.10.2019

Probenbezeichnung: lt. Kundeninformation:
Kompost 2S
Senecio Kompost
= A19.2882

Kunden-Proben-Nr.:			Kompost 2S Senecio Kompost
ÖHMI-Proben-Nr.:			
Kennzahl	Dimension	Methode	A19.2882
Trocken-rückstand	%	DIN EN 12880 Stand: 02-2001	53,7
Gesamt-Stickstoff	% OS	Methodenbuch VDLUFA	0,66
CAL-Extrakt		Methodenbuch VDLUFA	
Phosphor	kg/t OS	DIN EN ISO 11885 Stand: 09-2009	0,847
Kalium	kg/t OS	DIN EN ISO 11885 Stand: 09-2009	0,787
$CaCl_2$-Extrakt		Methodenbuch VDLUFA	
Ammonium-Stickstoff	kg/t OS	E DIN ISO 14256 Stand: 03-2003	0,0014

OS = Originalsubstanz

Bianca Denecke
Abteilungsleiterin Chemie

Die Prüfergebnisse beziehen sich ausschließlich auf das untersuchte Prüfmaterial. Sollte die Probenahme nicht durch die ÖHMI Analytik GmbH erfolgt sein, beziehen sich die Ergebnisse auf die Probe wie erhalten. Ohne schriftliche Genehmigung des Prüflaboratoriums darf der Prüfbericht nicht auszugsweise vervielfältigt oder veröffentlicht werden. Es gelten unsere Allgemeinen Geschäftsbedingungen. Mit Auftragserteilung gelten die Allgemeinen Geschäftsbedingungen als anerkannt.

Magdeburg,
16.10.2019

Prüfbericht

Prüfbericht-Nr.:
A19.2883

Auftraggeber: EXSEMINE GmbH
Am Wehr 4
06198 Salzatal OT Zappendorf

Email: g.horn@exsemine.de

Auftragnehmer / Ort der Labortätigkeit: ÖHMI Analytik GmbH
Berliner Chaussee 66
39114 Magdeburg

Probenahme: erfolgte durch Auftraggeber

Eingangsparameter: 14.10.2019 / 13:30 Uhr / 16,4 °C

Lagerung vor Untersuchung: Kühllagerung bei max. 5,0 °C

Untersuchungsbeginn: 14.10.2019 / 13:45 Uhr

Untersuchungsende: 16.10.2019

Probenbezeichnung: lt. Kundeninformation:
Kompost 3SL
Senecio und L.squarrosa
= A19.2883

Kunden-Proben-Nr.:			Kompost 3SL Senecio und L.squarrosa
ÖHMI-Proben-Nr.:			
Kennzahl	Dimension	Methode	A19.2883
Trocken-rückstand	%	DIN EN 12880 Stand: 02-2001	54,9
Gesamt-Stickstoff	% OS	Methodenbuch VDLUFA	0,70
CAL-Extrakt		Methodenbuch VDLUFA	
Phosphor	kg/t OS	DIN EN ISO 11885 Stand: 09-2009	1,07
Kalium	kg/t OS	DIN EN ISO 11885 Stand: 09-2009	0,908
$CaCl_2$-Extrakt		Methodenbuch VDLUFA	
Ammonium-Stickstoff	kg/t OS	E DIN ISO 14256 Stand: 03-2003	0,0015

OS = Originalsubstanz

Bianca Denecke
Abteilungsleiterin Chemie

Die Prüfergebnisse beziehen sich ausschließlich auf das untersuchte Prüfmaterial. Sollte die Probenahme nicht durch die ÖHMI Analytik GmbH erfolgt sein, beziehen sich die Ergebnisse auf die Probe wie erhalten. Ohne schriftliche Genehmigung des Prüflaboratoriums darf der Prüfbericht nicht auszugsweise vervielfältigt oder veröffentlicht werden. Es gelten unsere Allgemeinen Geschäftsbedingungen. Mit Auftragserteilung gelten die Allgemeinen Geschäftsbedingungen als anerkannt.

Magdeburg,
16.10.2019

Prüfbericht

Prüfbericht-Nr.:
A19.2884

Auftraggeber: EXSEMINE GmbH
Am Wehr 4
06198 Salzatal OT Zappendorf

Email: g.horn@exsemine.de

Auftragnehmer / Ort der Labortätigkeit: ÖHMI Analytik GmbH
Berliner Chaussee 66
39114 Magdeburg

Probenahme: erfolgte durch Auftraggeber

Eingangsparameter: 14.10.2019 / 13:30 Uhr / 16,4 °C

Lagerung vor Untersuchung: Kühllagerung bei max. 5,0 °C

Untersuchungsbeginn: 14.10.2019 / 13:45 Uhr

Untersuchungsende: 16.10.2019

Probenbezeichnung: lt. Kundeninformation:
Kompost 4L
Lapulla squarrosa
= A19.2884

Kunden-Proben-Nr.:			Kompost 4L Lapulla squarrosa
ÖHMI-Proben-Nr.:			
Kennzahl	Dimension	Methode	A19.2884
Trocken-rückstand	%	DIN EN 12880 Stand: 02-2001	54,1
Gesamt-Stickstoff	% OS	Methodenbuch VDLUFA	0,68
CAL-Extrakt		Methodenbuch VDLUFA	
Phosphor	kg/t OS	DIN EN ISO 11885 Stand: 09-2009	0,969
Kalium	kg/t OS	DIN EN ISO 11885 Stand: 09-2009	1,21
$CaCl_2$-Extrakt		Methodenbuch VDLUFA	
Ammonium-Stickstoff	kg/t OS	E DIN ISO 14256 Stand: 03-2003	0,0021

OS = Originalsubstanz

Bianca Denecke
Abteilungsleiterin Chemie

Die Prüfergebnisse beziehen sich ausschließlich auf das untersuchte Prüfmaterial. Sollte die Probenahme nicht durch die ÖHMI Analytik GmbH erfolgt sein, beziehen sich die Ergebnisse auf die Probe wie erhalten. Ohne schriftliche Genehmigung des Prüflaboratoriums darf der Prüfbericht nicht auszugsweise vervielfältigt oder veröffentlicht werden. Es gelten unsere Allgemeinen Geschäftsbedingungen. Mit Auftragserteilung gelten die Allgemeinen Geschäftsbedingungen als anerkannt.

Table S1
Calculated amounts of compost/press-cakes for each plot to meet nutritional requirements.

Region: Etzdorf (Saxony-Anhalt, Germany)
Plot´s area: 1.7 * 1.7 m² = 2.89 m²

Compost kind	Cultivated plant	kg/m²	kg/plot
Control-compost	*Triticum aestivum*	1.85	5.35
Senecio-compost	*T. aestivum*	1.47	4.25
Senecio/Lappula-compost	*T. aestivum*	1.52	4.38
Lappula-compost	*T. aestivum*	1.45	4.19
L. squarrosa press-cake	*T. aestivum*	0.33	0.96
Control-compost	*Hordeum vulgare*	0.93	2.68
Senecio-compost	*H. vulgare*	0.74	2.13
Senecio/Lappula-compost	*H. vulgare*	0.76	2.19
Lappula-compost	*H. vulgare*	0.72	2.09
L. squarrosa press-cake	*H. vulgare*	0.17	0.48

HPLC-ESI-MS/MS sum parameter analysis

HPLC-ESI-MS/MS sum parameter analysis was conducted using a 1200 Series HPLC (Agilent, Waldbronn, Germany) equipped with binary solvent delivery in combination with ESI-MS/MS triple quadrupole detection (3200 QTrap, Applied Biosystems MDS Sciex, Darmstadt, Germany).
The chromatographic separation was achieved using a 150 mm × 2.1 mm, 4 µm, Synergy Max-RP 80 Å column (Phenomenex, Aschaffenburg, Germany) including a pre-column cartridge of the same material. The following gradient was applied at a flow rate of 300 µL min^{-1} (solvent A: 0.3% formic acid in water, solvent B: 0.3% formic acid in acetonitrile): 0−2 min (95% A), 2−14 min (95−40% A), 14−15 min (40−0% A), 15−18 min (0% A), 18−19 min (0−95% A), 19−30 min (re-equilibration 95% A). Injection volume was 5 µL and the temperature of the column oven was set to 40 °C. Four transitions per compound (analyte and IST) were chosen.
For the analyte retronecine-diphthalate: 452.0 → 94.0 m/z (cell entrance potential (CEP), 18.0; collision energy (CE), 50), 452.0 → 120.0 m/z (CEP, 18.0; CE, 36), 452.0 → 149.0 m/z (CEP, 18.0; CE, 55) and 452.0 → 304.0 m/z (CEP, 24.0; CE, 40).
For the IST [9,9-$^{2}H_2$]-retronecine-diphthalate: 454.0 → 96.0 m/z (CEP, 18.0; CE, 50), 454.0 → 122.0 m/z (CEP, 18.0; CE, 36), 454.0 → 149.0 m/z (CEP, 24.0; CE, 55) and 454.0 → 306.0 m/z (CEP, 24.0; CE, 40).

Data analysis and integration was achieved with Analyst 1.6.2 Software (Applied Biosystems MDS Sciex, Darmstadt, Germany). The LOD/LOQ was 0.1 µg RE kg^{-1} and 0.3 µg RE kg^{-1}, respectively.

Identification and validation of the analyte/internal standard signals was achieved by monitoring retention time and co-occurrence of the ion transitions m/z 452.0 → 120.0 /452.0 → 94.0 m/z (analyte) and m/z 454.0 → 122.0 /454.0 → 96.0 m/z (IST). As additional control, the relative intensities of the transition in comparison to the IST were considered. The peak area of the analyte was corrected by subtracting 4% of the peak area of the internal standard, reflecting the amount of non-deuterated IST in the used IST preparation. The total PA/PANO content in retronecine equivalents (RE) was calculated using equation (1), representing the sum of 1,2-unsaturated retronecine/heliotridine-type PAs in a sample. To determine the result in the form of RE the known amount of IST (Mw 297.391 g mol^{-1}) was set in relation to the molecular weight of retronecine (Mw 155.197 g mol^{-1}), resulting in a RE factor of 0.52.

$$(1)\quad c\ [\mu g\ kg^{-1}]\ RE = \frac{\text{amount (IST) } [\mu g] \times 0.52 \text{ (RE factor)} \times \text{corrected area (analyte } m/z \text{ } 452.0 \rightarrow 120.0)}{\text{area (IST } m/z \text{ } 454.0 \rightarrow 122.0) \times \text{amount (liqueur/sample) [g/mL]}}$$

To convert sum parameter results (retronecine equivalents (RE)) into total PA-content the following approximation approach was conducted, e.g. if the major PA constituent was for example Lycopsamin-*N*-oxide (Mw = 315.36 g mol^{-1}) we applied a conversion factor of 2.032 to convert RE (Mw of retronecine = 155.197 g mol^{-1}) into a total amount of PA/PANOs in this sample.

Table S2 The detailed analysis results of extraction and profiling of the PAs of plant samples as Chemisch-physikalische Analyse (#45183) comprising 31 individual PAs and PANOs by QSI (Bremen, Germany).

sample name	details	sample kind	PA-Gehalt [µg/kg]	Lyc%	LycNOx%	Int%	IntNOx%
Convolvulus arvensis		shoot	4253.9	48.26	21.32	23.57	6.85
Echinochloa crus-galli	plant (2)	shoot	2944.2	64.56	6.23	27.67	1.54
Atriplex patula		shoot	2386.6	67.57	0	32.43	0
Viola arvensis		shoot	7254.7	50.83	13.9	31.33	3.94
L. multiflorum	50cm with vessel	shoot	38.1	70.18	2.12	27.7	0
L. multiflorum	50cm without vessel	shoot	335.1	72.09	0	27.91	0
L. multiflorum	200cm with vessel	shoot	8.6	66.03	12.74	21.23	0
L. multiflorum	200cm without vessel	shoot	21.4	65.93	13.92	20.15	0
L. multiflorum	400cm without vessel	shoot	8.3	77.78	0	22.22	0
T. aestivum	sowing stage (2020)	soil	17.4	0	100	0	0
H. vulgare	DC32	root	7.3	69.96	0	30.04	0
H. vulgare	DC33	shoot	38.7	72.88	5.93	21.19	0
Coriandrum sativum	sowing stage	soil	714.4	0	61.84	0	38.16
Pisum sativum	sowing stage	soil	111	5.92	85.71	2.86	5.51
Brassica napus	sowing stage	soil	332.7	0	63.22	0	36.78
H. vulgare	DC32	root	32.6	73.08	9.82	16.75	0.35
H. vulgare	harvesting time	soil	25.9	0	92.88	0	7.12
H. vulgare	harvesting time	root	44.8	54.13	3.13	42.74	0
T. aestivum	harvesting time, Plant 1	root	70.6	92.31	0	7.69	0
H. vulgare	harvesting time, Plant 2	root	397.7	66.18	0	33.82	0
T. aestivum	harvesting time, Plant 3	straw	66.1	53.2	17.25	28.76	0.79
H. vulgare	harvesting time, Plant 4	straw	41.8	59.86	4.93	35.21	0

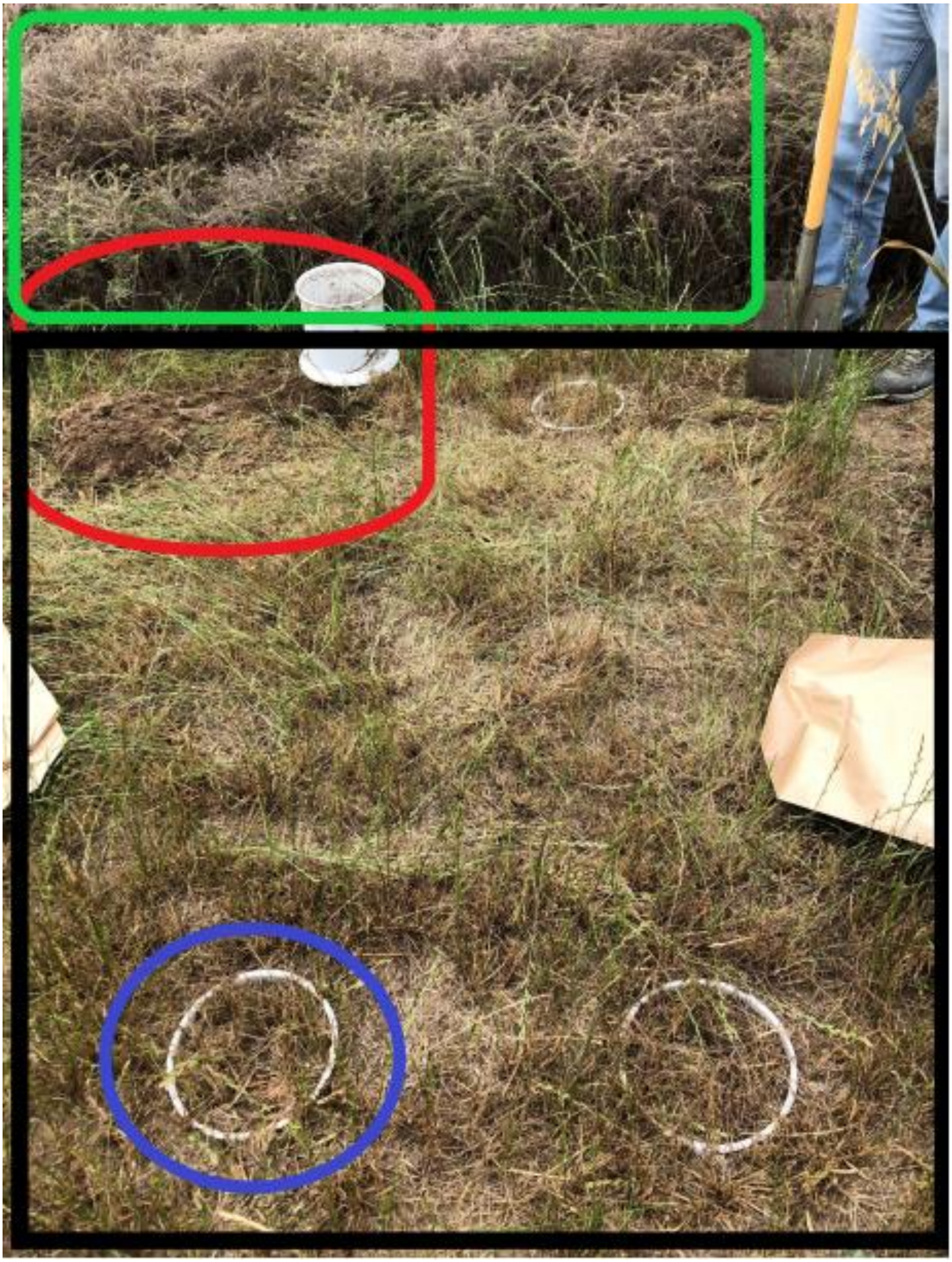

Figure S2. Picture to illustrate investigations of distance-related effects on PA-transfer. **Green**: *L. squarrosa* field; **Red**: Excavated Kick-Brauckmann vessel (50 cm) next to its hole; Black: field strip of *L. multiflorum*; **Blue**: Still buried Kick-Brauckmann vessel (200 cm) including *L. multiflorum*
